# Updated Lipoprotein(a) Genomic Risk Score and Aspirin for Primary Prevention of Cardiovascular Events

**DOI:** 10.1016/j.jacadv.2023.100754

**Published:** 2023-12-06

**Authors:** Chenglong Yu, George Thanassoulis, Rory Wolfe, John J. McNeil, Andrew M. Tonkin, Sotirios Tsimikas, Paul Lacaze

Elevated plasma lipoprotein(a) [Lp(a)] levels are an independent and likely causal risk factor for atherosclerotic cardiovascular disease (CVD). However, there are no pharmacological therapies currently approved for targeting elevated Lp(a). Aspirin is one of the most well-established therapies for secondary prevention of CVD and may be of benefit to individuals with elevated plasma Lp(a) in the primary prevention setting specifically.^1^^,^^2^

Plasma Lp(a) levels are largely genetically determined and heritable, meaning Lp(a)-associated genotypes can be used as a proxy to identify high-risk individuals in the absence of measured Lp(a) levels.^3^ Recently, Lacaze et al^2^ showed that individuals with elevated plasma Lp(a)-associated genotypes were more likely to benefit from aspirin in primary prevention and that the benefits of aspirin outweighed harms (bleeding risk) in this subgroup of the older population. The findings came from the ASPREE (Aspirin in Reducing Events in the Elderly) randomized trial of daily 100 mg aspirin versus placebo with a median 4.7 years follow-up, where an interaction was observed between the *LPA* gene variant rs3798220-C and aspirin (*P* = 0.049) for major adverse cardiovascular events (MACE). The study^2^ further examined the effect of an Lp(a)-genomic risk score (GRS) comprising 43 variants from within the *LPA* gene.^3^^,^^4^ This *LPA*-GRS was found to be associated with increased CVD risk in the placebo group of ASPREE, but the risk was attenuated in the aspirin group. However, no interaction was observed between the *LPA*-GRS as a continuous variable and aspirin allocation for MACE (*P* = 0.90).

In the present analysis, we sought to test whether an updated GRS (containing 110 plasma Lp(a)-associated variants, including new variants outside the *LPA* gene)^5^ interacted with aspirin in the same analyses of the ASPREE trial. Our hypothesis was that newly associated variants, including those outside the *LPA* gene, may provide further specificity to identify individuals who benefit from aspirin. We analyzed the same 12,031 unrelated (close relatives with a genetic relationship >0.05 were removed) ASPREE participants of non-Finnish European ancestry aged ≥70 years without prior CVD events at enrollment, using the same methods as described previously^2^ but testing the new GRS. The new score based on 110 independent (a stringent linkage disequilibrium r^2^ threshold <0.01 was used) genome-wide significant variants (*P* < 5 × 10^−8^) was reported in a recent genome-wide association study of plasma Lp(a) levels.^5^

Primary analysis involved testing the interaction between the updated GRS as a continuous variable (per SD) and categorical variable (low [Q1], medium [Q2-4], and high [Q5] groups) and aspirin allocation using a Cox proportional hazards model for incidence of MACE and clinically significant bleeding (CSB) events,^2^ adjusting for age, sex, and the top 3 principal components of genetic ancestry (consistent with previous studies).^2^^,^^3^ In secondary analysis, we tested for interaction between the 110 variants (minor-allele carrying status) and aspirin allocation individually using the same model.

The updated GRS, as a continuous variable, was associated with increased MACE risk in all participants (HR per SD of 1.10; 95% CI: 1.00-1.21; *P* = 0.043) in the model without interaction. This was consistent with our previous result using the previous 43-variant GRS.^2^ As a new finding, unlike the older GRS, we observed a significant interaction between the updated GRS as a continuous variable and aspirin allocation for MACE (interaction HR: 1.21 [95% CI: 1.00-1.47]), *P* = 0.046) (Table 1). The subgroup analyses for aspirin and placebo arms (Table 1) suggested benefit from aspirin therapy for those with higher Lp(a) levels genetically according to the new GRS. The interactions between the new GRS categorical groups and aspirin allocation were with larger effect sizes (interaction HR: 1.32 [95% CI: 0.78-2.21] for medium vs low GRS, and HR: 1.74 [95% CI: 0.95-3.19] for high vs low GRS) but not significant (*P* > 0.05) (Table 1), possibly due to smaller participant numbers in categorical subgroups, which may reduce power.

**Table 1:** Association of Lipoprotein(a) Genomic Risk Score With MACE Risk in the ASPREE Trial

Lipoprotein(a) GRS 110 Variants		All Participants (N = 12,031) MACE (n = 410)	Aspirin Arm (n = 5,974) MACE (n = 179)	Placebo Arm (n = 6,057) MACE (n = 231)
Continuous	Interaction (GRS ∗aspirin)	1.21 (1.00-1.47), *P* = **0.046**		
	GRS (per SD)		0.98 (0.85-1.14), *P* = 0.804	1.19 (1.06-1.34), *P* = **0.004**
Categorical	Interaction (medium vs low GRS ∗aspirin) Interaction (high vs low GRS ∗aspirin)	1.32 (0.78-2.21), *P* = 0.296 1.74 (0.95-3.19), *P* = 0.075		
	Low GRS (Q1) Medium GRS (Q2-Q4) High GRS (Q5)		Reference 0.88 (0.61-1.27), *P* = 0.487 0.97 (0.62-1.53), *P* = 0.903	Reference 1.17 (0.81-1.69), *P* = 0.403 1.69 (1.13-2.54), *P* = **0.011**

Values are HR (95% CI). **Bold** indicates a significant association (*P* < 0.05).

ASPREE = Aspirin in Reducing Events in the Elderly; GRS = genomic risk score; MACE = major adverse cardiovascular events; Q = quintile.

In secondary analysis, out of the 110 variants, 6 individual variants—rs9458011 (*PLG* gene), rs9457648 (*LOC101929142*), rs60227172 (intergenic), rs11970263 (*ZDHHC14*), rs78229704 (intergenic), and rs1247347 (*LOC102724087*)—all outside the *LPA* gene, were observed with interaction (testing minor-allele carrier status) with aspirin allocation for MACE (*P* < 0.05). While these results suggest the possibility that single-nucleotide polymorphisms outside the *LPA* gene may confer aspirin-specific effects, these associations did not remain significant after multiple testing correction, and therefore, should be interpreted with caution. Furthermore, compared to the original 43 variants within the *LPA* gene,^4^ the variants outside the *LPA* gene may have pleiotropic effects, which could introduce bias in the interaction effect. Further studies are warranted to investigate the potential pleiotropic effects of these variants.

We tested for interaction between the GRS and all above genotypes (new GRS or single variants) with aspirin allocation for bleeding (CSB) events, but found no interactions (all *P* > 0.05, data not shown), also consistent with our previous study.^2^

In conclusion, this analysis provides further evidence that aspirin may benefit older individuals with elevated Lp(a) genotypes in primary prevention of CVD events and that using an updated GRS containing Lp(a)-associated variants outside of the *LPA* gene may provide further specificity to detect an aspirin response. Further studies are warranted to replicate these findings in independent aspirin trials.
